# The role of National Immunisation Technical Advisory Groups (NITAGs) in strengthening national vaccine decision-making: A comparative case study of Armenia, Ghana, Indonesia, Nigeria, Senegal and Uganda

**DOI:** 10.1016/j.vaccine.2018.07.063

**Published:** 2018-09-05

**Authors:** Natasha Howard, Helen Walls, Sadie Bell, Sandra Mounier-Jack

**Affiliations:** London School of Hygiene and Tropical Medicine (LSHTM), Department of Global Health and Development, Tavistock Place, London WC1H 9SH, United Kingdom

**Keywords:** Vaccination, Vaccine policy, Low and middle-income countries, NITAGs

## Abstract

**Introduction:**

Improving evidence informed decision-making in immunisation is a global health priority and many low and middle-income countries have established National Immunisation Technical Advisory Groups (NITAGs) as independent technical advisory bodies for this purpose. NITAG development and strengthening has received financial and technical support over the past decade, but relatively little evaluation. This study examined NITAGs in six low and middle-income countries (i.e. Armenia, Ghana, Indonesia, Nigeria, Senegal, Uganda), to examine functionality, quality of recommendation development, and integration with national decision-making bodies and processes.

**Methods:**

A mixed-method case-series design, used semi-structured interviews, NITAG meeting observations, and document review. Data were analysed thematically.

**Results:**

Five NITAGs had been legally established with terms of reference and appeared well functioning, with Ghana’s in development. All NITAGs had standard operating procedures and nomination procedures to ensure a range of expertise, generally comprising 10–15 core, 1–5 secretariat, and several ex-officio members. Aside from economics, NITAGs reported a wide range of member expertise. Newer NITAGs had particular concerns about funding. Four used formal conflict of interest procedures, although some commented that implications were not always understood. NITAGs valued local data, and limited evidence suggested NITAG presence might reinforce data production through surveillance and local research studies. All observed meetings demonstrated due process and evidence-based decision-making processes were generally followed, with a critical role played by working-group data syntheses and assessments. NITAGs were seen as well integrated with ministry of health (MoH) decision-making and MoH interviewees were positive about NITAG contributions, indicating NITAGs had an important role. Collaboration with other bodies was more limited, but mitigated by NITAG members’ cross-membership in other bodies.

**Conclusions:**

NITAGs have an important and valued role within national immunisation decision-making. However, their position remains insecure, with the need for sustainable technical and financial support.

## 1 Introduction

Improving immunisation decision-making and national policy development through better evidence use is a global health priority [1]. Many low and middle-income countries (LMICs) have followed high-income countries in establishing National Immunisation Technical Advisory Groups (NITAGs). NITAGs provide independent technical guidance to national policy-makers and programme managers to support evidence-based and locally-relevant immunisation policy and programme decisions [2], [3]. Effective NITAGs are thus a global immunisation priority, and a marker of countries’ immunisation commitment, since the Global Vaccine Action Plan 2011–2020 called all countries to establish or have access to NITAGs by 2020 [1].

Since 2009, the World Health Organization (WHO) and Unicef included NITAG presence and six functionality indicators in annual immunisation Joint Reporting Forms (JRF): (i) formal written terms of reference; (ii) legislative or administrative basis for establishing the NITAG; (iii) core membership of at least five expertise areas (i.e. epidemiology, immunology, infectious diseases, paediatrics, public health); (iv) at least one meeting annually; (v) agenda and background materials distributed ahead of meetings; and (vi) declaration of interests by NITAG members [4], [5]. By late 2016, 125 of 194 WHO member states reported having a NITAG, 120 (96%) of which were officially legislated - including 22 low-income and 69 middle-income countries. Ninety (46%) reported their NITAG complying with all six JRF indicators, including 6 low-income and 46 middle-income countries [6].

Despite NITAGs’ recognised value for national decision-making [7], [8], relatively little independent analysis has been published. While several articles examined NITAG functioning and working processes, using data from international meetings and self-assessment, more can be learned from NITAGs and their challenges [9], [8], [10], [11], [12], [13]. As part of external evaluation of SIVAC (Box 1), a ten-year global initiative to develop and strengthen LMIC NITAGs [13], [14], [15], this study examines NITAG implementation, particularly functionality, quality, and integration in Armenia, Ghana, Indonesia, Nigeria, Senegal, and Uganda. Though Ghana’s NITAG was not yet established, it was included as a case because its development received considerable external support, particularly from SIVAC.

Box 1 **The SIVAC Initiative.**

**Table:** 

The SIVAC Initiative (2008–2017) provided technical support for establishing and strengthening NITAGs in low and middle-income countries [15]. Funded primarily by the Bill & Melinda Gates Foundation, through the Agence de Médecine Préventive (AMP) in collaboration with the International Vaccine Institute (IVI), the SIVAC Initiative provided some degree of support to 43 countries. It worked more extensively in 30 of these countries. Major SIVAC activities were advocacy, direct technical - and sometimes financial - support to countries, and development of training materials. Advocacy was a large component of SIVAC’s work, particularly during the initial years. SIVAC worked to raise the profile of NITAGs in the global agenda, through collaboration with academic institutions, participating in conferences and partner meetings, and publishing articles [12], [13].

## 2 Methods

### 2.1 Study design

We chose a mixed-methods case-series design, including semi-structured key informant interviews, meeting observations, and documentary analysis. Six case-study countries were selected based on LMIC status, feasibility (e.g. access, language, security), and to provide a range of: (i) regions, i.e. Africa, Asia, Europe; (ii) NITAG operation length; and (ii) eligibility for Gavi support, i.e. eligible, transitioning from support [16]. All cases received some SIVAC support to develop or strengthen capacity [13], [17].

### 2.2 Data collection

We recruited interviewees purposively, based on roles and knowledge, from NITAG core, secretariat, and ex-officio members (i.e. advisory members, by virtue of institutional position or affiliation – such as UN or NGO representatives, with no voting rights); government staff, including national immunisation programme and ministry of health (MOH) officials; and external partners involved in immunisation policy (e.g. WHO, Unicef). Most interviews were conducted face-to-face, during country visits, or via telephone and Skype. Interviewees provided written informed consent prior to interviews, which were audio recorded and professionally transcribed. We ensured anonymity by removing personal identifiers. We included 56 interviewees, i.e. 5 for Armenia, 8 for Ghana, 13 for Indonesia, 12 for Nigeria, 9 for Senegal, and 9 for Uganda during 2017 (Table 1).

**Table 1:** NITAG descriptions and recent recommendations.

NITAG	Status^a^	JRF 2017	Meetings and Working Groups (WGs)^b^	Examples of recent recommendations	NITAG background and description
Armenia Est. 2013	*Region:* EURO *Income:* L-MIC *Gavi:* Tran 2	Functional (5/6, except CoI process)	*Annual meetings:* 4 (2016) 4 (2015) 2 (2014) *WGs:* 2 (i.e. HPV, influenza) *Meeting observed:* June 2017	*Recommendation on HPV vaccine introduction (2017):* the NITAG supported the MoH Gavi application, based on consideration of the public health importance of cervical cancer in Armenia, data on efficacy and effectiveness and preliminary impact data from other countries. However, it recommended a demonstration project in 11 districts first to refine communications strategies and explore costs. *Developing national influenza vaccination strategy:* In response to MoH request, due to issues with changing funding levels and target populations.	Replaced a similar committee established in 2009. A 2015 evaluation indicated that it was functional, although not fully compliant with best practices (e.g. lack of CoI policy, lack of SOPs, no liaison or ex-officio officers, unclear agenda-setting process, unclear recommendation development framework). No MoH budget was allocated and operating costs were minimal. Armenia fulfilled all JRF criteria, except for members declaring conflicts of interest, since 2013. Four NITAG meetings were held in 2015 and five in 2016. Working groups were first established to review HPV vaccine introduction in 2016 and influenza vaccination policy in 2017.

Ghana Est: No	*Region:* AFRO *Income:* L-MIC *Gavi:* Tran 1	NA (0/6)	*Annual meetings:* None yet *WGs:* None *Meeting observed:* No	None	Began development in 2013. SIVAC, a global NITAG support initiative (2007–2017), conducted an assessment visit in late 2011 and supported development of a concept note issued in May 2013. Potential members were identified in 2015. However, political changes affected progress, with three minister of health between 2015 and 2017, disease outbreaks, lack of funding clarity, organisational divisions between the Ministry of Health and the Ghana Health Service, and confusion amongst some stakeholders about differences between NITAG and Inter-Agency Coordinating Committee (ICC).
Indonesian Technical Advisory Group on Immunisation (ITAGI) Est. 2007	*Region:* SEARO *Income:* L-MIC *Gavi:* Tran 2	Functional (6/6)	*Annual meetings:* 3 (2016) 7 (2015) 3 (2014) *WGs:* As needed *Meeting observed:* April 2017	*Recommendation on dengue vaccine introduction (2018):* Responding to a 2016 MoH request on the new dengue vaccine, ITAGI began discussions and expect to make a recommendation in early 2018. *Recommendation on Measles-Rubella vaccine introduction (2016):* ITAGI recommended that MoH implement a catch-up campaign of rubella vaccination combined with measles vaccination, targeting 9mo-15y, followed by routine immunisation for all infants. *Recommendation on Japanese Encephalitis vaccine in Bali Province (2016):* ITAGI recommended that MoH introduce JE vaccine with a ‘crash programme’ in one of the most endemic and strategic provinces, with the most complete JE data on JE, and use results to develop strategic plans in 3 other high endemicity provinces.	The oldest, it was established as an independent advisory committee to the national immunisation programme and MoH, replacing another expert committee performing a similar function. It has fulfilled all JRF criteria since recording began in 2010. It consists of approximately 3 secretariat and 18 core members from a range of disciplines. It has comprehensive SOPs and holds 4 meetings annually, plus provision for more frequent smaller meetings. Funding comes from MOH, Gavi, and a small amount from WHO. Meeting travel and accommodation are covered, but staff are unpaid with the exception of one support person.

Nigerian Immunisation Technical Advisory Group (NGI-TAG) Est. 2015	*Region:* AFRO *Income:* L-MIC *Gavi:* Tran 1	Functional (6/6)	*Annual meetings:* 4 (2017) 0 (2016) 2 (2015) *WGs:* 7 (i.e. cerebrospinal meningitis, polio, measles, rotavirus, HPV, tetanus, yellow fever) *Meeting observed:* Sep 2017	*Recommendation on introducing meningitis vaccine (2017):* MoH requested a recommendation on whether and which meningitis vaccine to introduce into routine immunisation. After review and discussion of WG findings, NGI-TAG recommended the introduction of MCYW vaccine by 2022, and in the interim to use MenAfriVac as less effective but more affordable for routine introduction.	The youngest, inaugurated on 17 August 2015, by the Permanent Secretary of the Federal Ministry of Health (FMOH), and is hosted by the National Primary Health Care Development Agency, a parastatal of Nigeria’s FMOH. The NGI-TAG developed comprehensive standard operating procedures (the “Green Book”) and conflict of interest procedures and aims to meet quarterly. Initial lack of funds prevented NGI-TAG or working group meetings until 2017. The NITAG has fulfilled all JRF criteria since 2015. Seven vaccine working groups were initially organised for cerebrospinal meningitis, polio, measles, rotavirus, human papilloma virus, tetanus, and yellow fever. SIVAC supported training of some members through a workshop and vaccinology course and on-the-job guidance.

Senegal Comite Consultatif pour la Vaccination au Senegal (CCVS) Est. 2013	*Region:* AFRO *Income:* LIC *Gavi:* Tran 1	Functional (6/6)	*Annual meetings:* 3 (2016) 3 (2015) 3 (2014) *WGs:* 2–3, as needed *Meeting observed:* April 2017	*Recommendation on introducing birth-dose Hepatitis B vaccination:* The recommendation to introduce the Hep B vaccine at birth and adopt the WHO recommendation by giving a longer time window to vaccinate – within 72 h with a view to reach out to children that are not delivered in health care facilitate, and prepare a tailored introduction plan for each district *Recommendation on introducing Menafrivac vaccine into routine immunisation:* Analysis of local epidemiological data, including organising a national surveillance workshop, showed that after 2012 and 2013 mass campaigns, MenA cases virtually disappeared while numbers of other valents grew steadily. As Senegal will transition from Gavi support soon, CCVS recommended, after extensive discussion, to introduce 1 dose at 15mo, invest in strengthening surveillance, review in 2019 and consider shifting to a quadrivalent vaccine.	Senegal’s NITAG was established approximately two years after initial development discussions (Jan 2012). The rationale described in official documents mention improving the quality of the decision making process and the need to “take better account of local specifics of vaccination”. The NITAG’s scope was wide ranging, aiming to oversee and monitor immunisation policy and programme, provide technical and scientific expertise, establish partnerships with national and international bodies, and advocate for immunisation. The NITAG has fulfilled all JRF criteria since 2014. A review of 2014–2016 work-plans showed that a majority of technical activities were implemented, notably those related to specific vaccine recommendations. Some recommendations were postponed, e.g. strategic review of the immunisation programme and a review of the NITAG sustainability strategy were postponed because of challenges in organising meetings, unexpected priorities (e.g. Ebola), and lack of secretariat staff availability.

Ugandan National Immunisation Technical Advisory Group (UNITAG) Est. 2014	*Region:* AFRO *Income:* LIC *Gavi:* Eligible	Functional (6/6)	*Annual meetings:* 4 (2016) 3 (2015) 0 (2014) *WGs:* 5 (i.e. new vaccine priorities, MenA, rotavirus, influenza, malaria) *Meeting observed:* April 2017	*Recommendation on introducing Rotavirus vaccine (2015):* The UNITAG WG presented a technical report to a plenary of members in August 2014 and following discussion, core members submitted the consensus opinion that Rotavirus vaccine should be introduced into Uganda’s routine immunisation program subject to availability of sustainable government financing. *Recommendation on introducing MenAfriVac vaccine (2015):* UNITAG recommended that MenAfriVac vaccine be limited to campaigns in high-risk districts in Northern and Western Uganda and that proposals for introduction into routine immunization or catch-up campaigns be considered separately.	Replaced the Ugandan Advisory Committee on Vaccines and Immunization (ACVI), a similar body established in 2012. ACVI formation was spearheaded by the Uganda National Academy of Sciences (UNAS), which became UNITAG Secretariat. UNITAG aims are wide ranging, including providing technical and scientific expertise to the government and advising national immunisation policy and programme implementation. It has fulfilled all JRF criteria since 2015.

NB: ^a^Gavi eligibility, LMIC status, and JRF status were assessed for 2017; ‘Tran 1’ and ‘Tran 2’ refer to Gavi’s funding transition phases [23]. ^b^’Meetings’ refers to numbers or average number of annual meetings; ‘WGs’ refers to number of working-groups; ‘Observed’ refers to if and when the NITAG was observed.

We observed NITAG meetings in five countries. Ghana’s NITAG was not yet established and thus not visited. The observation template was designed to ensure inclusion of relevant issues (e.g. members and roles, evidence appraisal, decisions) while including emergent issues. Meetings were conducted in English, except in Armenia and Indonesia, which were translated professionally and notes taken simultaneously.

We reviewed technical reports, administrative documents (e.g. NITAG Standard operating procedures, meeting minutes), and NITAG outputs (e.g. recommendations).

### 2.3 Analysis

We analysed data thematically using *NITAG Evaluation Tool* categories [18]: (i) functionality; (ii) quality of processes and outputs; (iii) integration with national decision-making (Table 2). We did not evaluate individual NITAGs, instead examining NITAG roles, challenges, and achievements overall.

**Table 2:** NITAG assessment^a^ criteria, categories, challenges^b^, and findings.

Criteria	Description	Assessment categories	NITAG challenges identified by WHO-SAGE, April 2017^b^	Case study findings (excluding Ghana)
Functionality	Functional NITAG structure and operations, fostering timely generation of recommendations.	• *Structural viability* (i.e. whether NITAG has been legally established with specific TORs and guaranteed resources for routine functioning)	• Lack of secretariat; • Funding concerns;	• Most were functional, with specific TORs; • Some had funding sustainability concerns (e.g. secretariat funding); • Willingness to expand TORs to programme steering, sustainability assessment, and issues beyond vaccine introduction.
		• *Functional capacity* (i.e. having and complying with formalised and approved SOPs, member nomination procedures, and areas of expertise; whether activity planning and execution include formal work-plans and working groups; whether NITAG has formal reporting obligations to MoH; existence and use of a formal COI policy, and consequences of declared interests.)	• Lack of SOPs and work-plans; • Instability; • Inadequate procedures (e.g. COI).	• All had approved SOPs and work-plans; • Less-supported NITAGs (e.g. lacking technical support/training) appeared less functional/sustainable; • Reporting obligations to MoH varied; • Four had formal COI procedures, though one reported implementation challenges; • No consequences of declared interests were observed.
		• *Productivity* (i.e. number of recommendations issued, whether these were part of work-plans and within expected timeframes, and how NITAGs responded to any urgent MoH requests)	• Lack of annual work-plans.	• Variation in numbers of recommendations produced and taken up by MoH, largely due to differences in maturity and funding levels.

Quality	NITAG capacity and quality of data collection, analysis, and synthesis processes, evidence and data needed to deliver recommendations.	• *Human resource capacity* (i.e. whether secretariat have necessary technical skills; members have opportunities to build their capacity to use scientific evidence; the NITAG is able to access external technical expertise, international and national scientific data as needed; and how working-groups are mandated and coordinated, members nominated, and outputs reported)	• Lack of expertise and training.	• Secretariat training and technical skills varied, depending largely on external support levels; • Evidence of access to technical expertise and scientific data, to different degrees; • Working-groups were operational and produced evidence-based reports, though numbers, mandate, coordination, and member nomination procedures varied.
		• *Analytical process quality* (i.e. whether and how members apply specific frameworks to defining policy issues, research questions, type/importance of data, and data collection; how members synthesise evidence and assess quality; and how recommendations are decided)	• No formalised processes.	• Formal processes existed and appeared to be followed.
		• *Outputs quality* (i.e. whether recommendations include a standardised summary of development process and technical questions addressed)	• NA	• Evidence of good quality reviews, particularly among NITAGs that received external training.

Integration	Integration and recognition within the national decision-making system.	• *Transparency* (i.e. whether governing policies were publically available; non-member participation; and how stakeholder concerns were addressed)	• NA	• NITAG governance policies and documentation were available, though not all NITAGs had their own web presence, and some used the NITAG Resource Centre; • Non-member observation was supported; • Unclear how stakeholder concerns addressed.
		• *Interaction with national stakeholders* (i.e. frequency, format, channel, formality, and focal person for communication with MoH, stakeholders, and public; national immunisation collaborators and perceived antagonisms; and participation in relevant fora)	• External partner processes not involving NITAG; • NITAG activities not in country plans.	• Interactions with other bodies (e.g. ICC) varied and were mostly informal; • Integration was an area in which most NITAGs required further progress.
		• Acknowledgement by nationally-relevant parties (i.e. awareness of NITAG existence and role by decision-makers, implementers, and public; take-up of recommendations by MoH; whether members were called as resources; and whether other organisations disseminated NITAG recommendations)	• Confusion with other bodies (e.g. ICC, HTA); • Low awareness of NITAG role.	• Recommendations issued and reportedly adopted by MoH; • Members were considered national experts and called as resources, though it was unclear whether this related to NITAG affiliation; • Some NITAGs had recommendations disseminated.

NB: ^a^Evaluation framework adapted from [18]. ^b^Included a broad range of NITAGs [24].

### 2.4 Ethics

The London School of Hygiene and Tropical Medicine Observational Research Ethics Committee provided approval (reference 12036).

## 3 Results

### 3.1 Functionality

*NITAG structural viability*: All five NITAGs had been legally established with developed terms of reference (TORs). In addition to advising MoH on new vaccines, some interviewees suggested expanding NITAG TORs to advising national vaccination programmes (e.g. Indonesia, Senegal, Uganda) on technical and cost-effectiveness issues, with one describing the NITAG as a ‘think-tank’ for the vaccination programme.

“...if we start [vaccine implementation] and then funding becomes a problem, you’ll find you have started on something which you cannot carry on, and this is the big role that we have given NITAG to advise us...” (Uganda#5)

While Ghana was slow to establish a NITAG, challenges to national vaccination programme sustainability appeared to generate renewed interest in its NITAG, after years of delay.

“I believe that the government, the minister, and the ministry are very interested in setting-up the NITAG, and especially so with the transitioning from Gavi support” (Ghana#5)

Other NITAG roles included providing credibility, raising public immunisation awareness, engaging with healthcare professionals, and acting as referee or technical resource in response to rumours or hesitancy. For example, ITAGI (Indonesia) regularly developed recommendations addressing community anti-vaccine sentiment. Nigerian interviewees noted that a NITAG could have avoided or mitigated the ‘polio controversy’ [19]. A positive initiative for UNITAG (Uganda) was contributing to the passing of a national immunisation law. A crucial NITAG role was in emphasising a shift to transparent, impartial and inclusive decision-making from reliance on a few expert opinions for national immunisation programmes.

Newer NITAGs (e.g. Nigeria, Uganda, Senegal) expressed concerns about lacking guaranteed funding, while all advocated increasing the reliable funding of secretariats. Established NITAGs (e.g. Armenia, Indonesia) relied predominantly on government funding for running costs, which were kept relatively low (e.g. member time was donated, capacity-building and international travel was minimal and/or externally funded). NITAGs shared challenges in mobilising sustainable government and/or external funding. Ensuring funding mechanisms did not jeopardise NITAG independence was a concern identified in some countries (e.g. Nigeria). Several had received significant external operational support – through, for example, SIVAC or WHO, for funding of secretariat, meetings, and/or trainings (e.g. Indonesia, Senegal, Uganda), joint work-plan development, or development of specific recommendations (e.g. Nigeria, Senegal, Uganda), raising concerns about sustainability (e.g. Nigeria, Uganda, Senegal) after this support ended. Some CCVS (Senegal) members indicated that given minimal support of basic running cost by government and/or WHO and additional training on evidence review and vaccinology, it could continue to work effectively. UNITAG members acknowledged that Uganda’s MoH should take more ownership of UNITAG funding, but given MoH constraints and priorities, this was not considered an immediate solution to anticipated needs for long-term financial and capacity-building support.

“Members operate on a three year tenure and our first tenure is expiring at the end of this year, so we’re going to get maybe 40% new members starting next year and the whole process has to start again to try and get them to understand what needs to be done.” (Uganda#3)

*Functional capacity:* All NITAGs had Standard Operating Procedures (SOPs) and nomination procedures to ensure a relevant range of expertise. NITAGs generally comprised 10–15 core members (including chair and deputy), 1–5 secretariat, and several ex-officio members. Core members covered the five JRF expertise areas (i.e. epidemiology, immunology, infectious diseases, paediatrics, public health) and identified specialities, e.g. microbiology, neurology, and health policy. Clinical specialties dominated membership. The Secretariat, often seconded from national immunisation programmes, provided administrative and technical support and linkages with MoH. Ex-officio observers included MoH and donor and technical partners (e.g. WHO, Unicef, Sabin). Thus, NITAG meetings could have 20–30 people attending. Members’ professional recognition and expertise was valued because of technical benefits but also as reinforcing NITAG credibility nationally. Those operating for several years indicated members had a good understanding of how to conduct activities and deliver recommendations. All NITAGs reported an appropriate range of expertise, with the exception of economic evaluation, and in one case legal expertise, as members were generally less familiar with these technical areas and they were not included as expectations in JRF reporting.

All NITAGs reported that planning included developing annual work-plans. Four NITAGs had working-groups of 5–7 members developing specific recommendations (Table 1). The Armenian NITAG, which had received minimal external support, was reportedly still strengthening operating procedures and initiating working-groups for specific vaccines (e.g. HPV, influenza) [20]. While most NITAGs had reporting obligations, the format and formality of these varied.

Most NITAGs used formalised conflict of interest (COI) procedures, although some commented that implications were not always understood.

“We use it [COI form] but whether we give it the attention it deserves I think is another matter. I think many people, just as a knee-jerk reflex may sign not really understanding the full implication of what they have done by signing the form.” (Nigeria#7).

Actual consequences of declaring interests remained unclear, as no interviewees described examples of interests being declared.

*Productivity:* Table 1 shows recent recommendations. Work-plan agendas were either set entirely by MoH (e.g. Nigeria, Senegal, Uganda) or in collaboration with NITAGs (e.g. Armenia, Indonesia), which were generally very responsive to MoH requests. Time-frame issues remained unclear, as several NITAGs were new and financially constrained in the years preceding the study. However, all reported issuing recommendations within agreed timeframes (e.g. Nigeria issued its first in 2017).

### 3.2 Quality

*Human resources capacity and data access:* A strong secretariat was considered essential to NITAG functioning. As several NITAGs relied on secretariat who were seconded part-time from national programmes, capacity and relevant technical skills were sometimes problematic. Member opportunities to strengthen their capacity to collect, synthesise and analyse evidence in decision-making also varied, primarily on the amount of external technical support available.

NITAGs valued local data, and limited evidence suggested NITAG presence might reinforce data production through surveillance and local research studies. NITAG members noted that local data might not always be available or of sufficient quality for decision-making, and were generally pragmatic about using the best-quality data that was accessible. Members typically used university and hospital affiliations, and/or links with local partners, to source non open-access literature and local evidence. Using local data and context to tailor WHO-SAGE recommendations was identified as a key value of NITAGs. For example, Armenian interviewees noted the importance of reviewing local influenza vaccination target groups, as these were often modified depending on funding source (i.e. external, governmental), leading to health-worker confusion.

“We prefer the local data of course, because the local data explain the characteristics of our country. So, that’s why we are strengthening the surveillance.” (Indonesia#23)

“Senegal has adopted the recommendation to suit its local context and ensure better feasibility. The WHO was recommending 24 hours [for HepB birth-dose] but Senegal decided to advise vaccination within 72 hours of birth [as most births occurred at home]... The role of the committee is to make sure that the WHO recommendation is applicable in Senegal.” (Senegal#8)

While working-group mandates were relatively consistent, coordination, nomination, and output reporting could be further systematised.

*Analytical process quality:* All meetings observed showed a high degree of due process and, despite some weaknesses, that an evidence-based decision-making process was followed, with a critical role played by working-group data syntheses and assessments. Plenary discussions were observed as very active and of generally good quality. Evidence from working-group documentation (e.g. Uganda, Indonesia, Senegal) showed that literature was extensively reviewed, and local data and cost implications integrated into analyses. Several NITAGs used new vaccine ‘piloting’ to gather additional evidence, notably on cost-effectiveness, before scaling-up nationally (e.g. Armenia, Indonesia). However, all but UNITAG and ITAGI indicated limited consideration of economic evaluation in decision-making, a particularly important issue in countries approaching Gavi transition (e.g. Armenia, Nigeria).

*Outputs quality:* NITAGs had varying degrees of standardisation of recommendations, with UNITAG producing particularly well-structured outputs. This included variation in publication of review and evidence synthesis results, with not all routinely disseminating the scientific rationale behind their recommendations.

### 3.3 Integration

*Transparency:* While most NITAG members appeared willing to share governance documents, most did not have websites, though some shared recommendations through the NITAG Resource Centre (http://www.nitag-resource.org/). All allowed non-member observers by prior arrangement. Methods of addressing stakeholder concerns varied, depending on the nature of concerns (e.g. policy, technical, role) and was often through advising MoH.

*Interaction with national decision-makers and stakeholders:* Interaction format and channels varied, but were usually through chairpersons and/or secretariat. Interviewees noted collaboration with national decision-making bodies (e.g. ICC - interagency coordinating committee, national regulatory authority) as important, but not always well defined or understood, and sometimes reflecting a fragmented institutional landscape. This was potentially worsened by parallel committees overseeing vertical programmes (e.g. polio national certification committees) and sometimes mitigated by NITAG members’ cross-memberships in other bodies (e.g. Senegal, Uganda). In countries without ICCs (e.g. Indonesia), it was noted that NITAGs had a more pivotal technical role.

*Acknowledgement by national parties*: Successful and mature NITAGs were more fully integrated within national decision-making processes and valued by MoH. MoH interviewees were generally very positive about NITAG contributions, indicating it had an important role. However, interviewees acknowledged that NITAGs operate within political environments (e.g. resources available, public vaccine demand, presence of local manufacturers) and that MoH based decisions on factors additional to research evidence. MoH acknowledgement and awareness of NITAG roles/responsibilities appeared limited in Armenia and Ghana, both Gavi transitioning countries. In some case, NITAGs provided technical support for MoH to resist political pressures to introduce new vaccines, e.g. due to costs and/or effectiveness limitations.

“I know the ITAGI is already good [...] They always ask, they try to understand. If they don’t understand, they will say ‘OK, we need time to understand this’. And then they will ask questions to make them understand, and then they will review some more evidence, if required. So it is back and forth, back and forth. Only for immunisation is evidence-based decision-making process working, because of the ITAGI.” (Indonesia#24)

Members and MoH alike indicated that recommendations were considered, although funding was not always available for implementation. For example, Armenia produced 4–5 recommendations in the past two years, all of which were adopted by MoH. Indonesia produced 5, which were also successfully adopted. Senegal and Uganda each produced 3, some of which have already been accepted, and Nigeria recently produced its first recommendation. However, NITAGs sometimes produced recommendations for which MoH decisions had already been made, e.g. to submit a Gavi application. Integration within MOH processes was seen as essential by NITAG members, not only to ensure that evidence-based decision-making could be built into the process but also because this contributed to building recognition and motivation for NITAG members and brought credibility to the whole vaccination programme.

“Without the committee, they would have to ask individual experts, and this would be less independent and formalised. It would also be less efficient. For example, it took years for one person to advocate for the introduction of HepB birth-dose, including lobbying the first lady [...]. With the committee it had more weight.” (Senegal#1)

NITAGs were described by interviewees as an instrument of ownership at country level. In several examples, NITAGs capacitated countries to respond to partner ‘offers’ (e.g. of funding for specific vaccines) based on local evidence and needs. This was identified as increasing ability to push back against ‘felt pressure’ from manufacturers and funding partners.

“WHO was pushing us at country level to introduce the [Menafrivac] vaccine, but the CCVS said “No, let’s wait”.... They said serotypes and vaccines were not aligned.” (Senegal#6)

## 4 Discussion

NITAGs were described as instruments of country ownership, which could use evidence to tailor immunisation investments to country-specific epidemiology and health systems, thus enhancing financial sustainability. They were perceived as helping resist external pressures to rapidly introduce less relevant or unsustainable vaccines and described in several countries as examples of good practices that other national programmes should use. The importance of national ownership of vaccine introduction reflected findings from Ba-Nguz *et al*, who described it as “*paramount to the credibility and sustainability of immunisation programmes*” [11]. Challenges to functionality were similar across NITAGs, relating to the difficulty of mobilising unpaid time-constrained experts, resource constraints, insufficient good-quality local data, sometimes insufficient integration with national decision-making bodies, and comparable with findings by Adjagba et al. [12], [21]. However, each NITAG was situated in a specific political, economic and cultural context. For example, Ghana highlighted political challenges in establishing a NITAG, while Indonesia, a majority Muslim country with strong community anti-vaccine sentiment, highlighted socio-cultural challenges in addressing local sensitivities around vaccine use.

Findings showed that despite challenges, NITAGs were able to function satisfactorily in LMICs and provided valuable contributions to evidence-based decision-making. With adequate technical support, these NITAGs have gradually progressed from providing ‘expert opinion’ to a formalised and transparent decision-making process, a key purpose of NITAGs that is increasingly valued by health ministries. In countries transitioning from Gavi support (e.g. Ghana, which is currently struggling with planning for continuing to fund a large portfolio of vaccines without Gavi support), a well-functioning NITAG might help provide the relevant evidence for programme sustainability. Findings also indicate that NITAGs’ role could extend over time, from steering and reviewing the overall immunisation programme, to addressing vaccine confidence issues and harnessing local research to produce needed context-specific data.

Findings were based on participant perceptions, and non-interviewees may have held different views. While the six cases represent a range of regions and countries, each NITAG exists in a particularly context and results cannot be directly generalised. Despite this, findings were generally similar, contributing to knowledge on NITAG functionality, process quality, and national integration.

While NITAGs have an important and valued role within national immunisation decision-making [22], their position remains somewhat insecure, requiring ongoing technical and in some cases financial support.

## Conflict of interest

None declared.

## Author contributions

NH drafted the manuscript, with inputs from HW and SMJ. NH and SMJ conceived the study. NH, SMJ, and HW collected data. NH, HW, and SB coded and analysed data. SMJ provided critical review. All authors approved the version for submission.
